# Burkitt Lymphoma Presenting as Acute Abdomen: Beyond Infection and Diverticulitis

**DOI:** 10.7759/cureus.98608

**Published:** 2025-12-06

**Authors:** Catarina Faria Tavares, Catarina Maia, Catarina Sousa-Lopes, Tânia Martins, Catarina Carvalho, Ana Lúcia Cardoso

**Affiliations:** 1 Pediatrics Department, Unidade Local de Saúde de Viseu Dão-Lafões, Viseu, PRT; 2 Pediatric Cardiology Department, Unidade Local de Saúde de Santo António, Porto, PRT; 3 Pediatric Surgery Department, Unidade Local de Saúde de Santo António, Porto, PRT; 4 Pediatric Intensive Care Unit, Unidade Local de Saúde de Santo António, Porto, PRT

**Keywords:** acute abdomen, adolescent, burkitt lymphoma, meckel’s diverticulitis, peritonitis

## Abstract

Burkitt lymphoma (BL) is a highly aggressive mature B-cell non-Hodgkin lymphoma and a clinically important entity in pediatric oncology. In the sporadic form, abdominal involvement is frequent and may present with nonspecific gastrointestinal symptoms that can mimic common infectious or surgical conditions. However, presentations mimicking other acute surgical conditions, including Meckel-related complications with secondary peritonitis, are exceedingly rare and described almost exclusively in isolated case reports. We report the case of a previously healthy adolescent who presented with acute abdominal pain initially suggestive of a benign or infectious etiology, which subsequently evolved to a clinical picture compatible with Meckel’s diverticulitis and peritonitis. Progressive clinical deterioration prompted extended imaging and surgical exploration, which ultimately led to the diagnosis of sporadic Burkitt lymphoma and the prompt initiation of chemotherapy, with rapid clinical improvement. This case illustrates the diagnostic challenges posed by atypical abdominal presentations of Burkitt lymphoma in older children and underscores the importance of including lymphoproliferative disease in the differential diagnosis of adolescents with persistent or unexplained abdominal symptoms in order to facilitate earlier recognition and optimize outcomes.

## Introduction

Sporadic Burkitt lymphoma (BL) is the most common subtype of B-cell non-Hodgkin lymphoma in children and is characterized by a translocation involving the *MYC* oncogene, most frequently t(8;14)(q24;q32) [1]. BL is also one of the fastest-growing human tumors, with a doubling time of approximately 24 hours [2]. Non-Hodgkin lymphomas represent 7% of all pediatric cancers, of which BL accounts for more than 40% [1,2].

Three clinical variants of BL are recognized, each with distinct features. The endemic type, common in malaria-endemic regions, is strongly associated with Epstein-Barr virus (EBV) infection and typically involves the jaws. The sporadic type predominates in non-endemic areas such as North America, Europe, and East Asia and most often presents with abdominal, nodal, or genitourinary involvement. The immunodeficiency-associated variant occurs in patients with HIV/AIDS, posttransplant immunosuppression, or congenital immunodeficiencies [2].

Gastrointestinal symptoms are common, as they frequently present with abdominal symptoms, with abdominal pain occurring in roughly 60%-80% of cases due to rapid tumor expansion in the ileocecal region. Abdominal distension is also common, reported in about 40%-60% of children, and often reflects a large intra-abdominal tumor burden or ascites. A palpable abdominal mass is found on clinical examination in approximately 50%-70% of patients, typically firm and non-tender. Gastrointestinal symptoms such as vomiting, altered bowel habits, or signs of intestinal obstruction occur in 20%-40% of cases, sometimes presenting as intussusception. On clinical examination, additional findings may include hepatomegaly or splenomegaly (in about 20%-30% of patients) and abdominal tenderness, reflecting disease spread or local inflammatory effects [1-4].

In the abdomen, BL typically arises in the ileocecal region (particularly the terminal ileum, cecum, and appendix), reflecting the abundance of lymphoid tissue in this area [3]. While abdominal involvement is common, presentations mimicking other acute surgical conditions are rare. Reports of BL presenting as Meckel’s diverticulitis with secondary peritonitis are exceptionally uncommon [4]. Because of the rarity, this presentation represents well under 1% of pediatric Burkitt lymphoma cases, appearing almost exclusively in isolated case reports rather than epidemiological series.

Such presentations may mask the underlying hematologic malignancy, delaying diagnosis and appropriate management. Maintaining a high index of suspicion in cases of unexplained intra-abdominal inflammation or obstruction is crucial, as the early recognition of BL allows for the timely initiation of intensive chemotherapy, which is critical for improving outcomes.

## Case presentation

A previously healthy 17-year-old adolescent presented to the emergency department (ER) with a six-day history of pollakiuria, bladder tenesmus, and suprapubic pain, associated with anorexia. He stated that he had no fever, weight loss, or night sweats.

On examination, he had suprapubic tenderness without signs of peritoneal irritation. Laboratory tests showed a C-reactive protein (CRP) of 70 mg/L and a full blood count with normal leukocytes. A urine culture was collected and later returned negative, and urinalysis showed low-grade leukocyturia, but nitrite was negative. Empirical outpatient therapy for lower tract urinary infection was initiated with oral amoxicillin-clavulanate.

Three days later, he returned with worsening abdominal pain, now radiating to the lower anterior thorax, accompanied by asthenia and persistent anorexia. He denied nausea, vomiting, fever, respiratory symptoms, dysuria, altered bowel habits (including upper or lower gastrointestinal bleeding), or weight loss. There were no relevant epidemiological contacts. On examination, vital signs included a temperature of 36.8°C, a blood pressure of 124/82 mmHg, and a resting heart rate of 98 beats per minute. On abdominal examination, the lower quadrant presented tenderness with guarding. No masses were palpable, and bowel sounds were present. Rectal examination was not performed. No peripheral lymphadenopathy or edema was noted. No other systemic findings were observed, namely, rashes.

**Figure 1:**
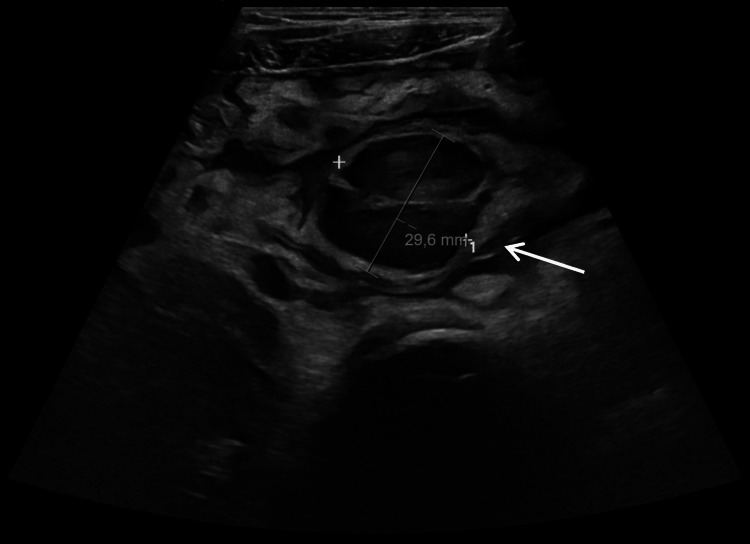
Abdominal ultrasound: hypoechoic nodular structure measuring 30 × 21 mm in the hypogastrium, suggestive of Meckel’s diverticulitis. The arrow indicates the suspected Meckel-related mass.

Chest radiography, ECG, and echocardiography were unremarkable. Laboratory tests showed a further increase in CRP (110 mg/L), with no other relevant abnormalities. Abdominal ultrasound revealed peritonitis, with moderate intraperitoneal fluid, diffuse peritoneal thickening with mesenteric fat stranding, and bowel wall thickening, together with a non-compressible hypoechoic nodular structure measuring 30 × 21 mm in the hypogastrium, corresponding to the site of maximal tenderness, suggestive of Meckel’s diverticulitis (Figure 1). The appendix was not clearly visualized, and small lymphadenopathies were observed in the nearby region, none with radiological findings of suspected malignant etiology.

**Figure 2:**
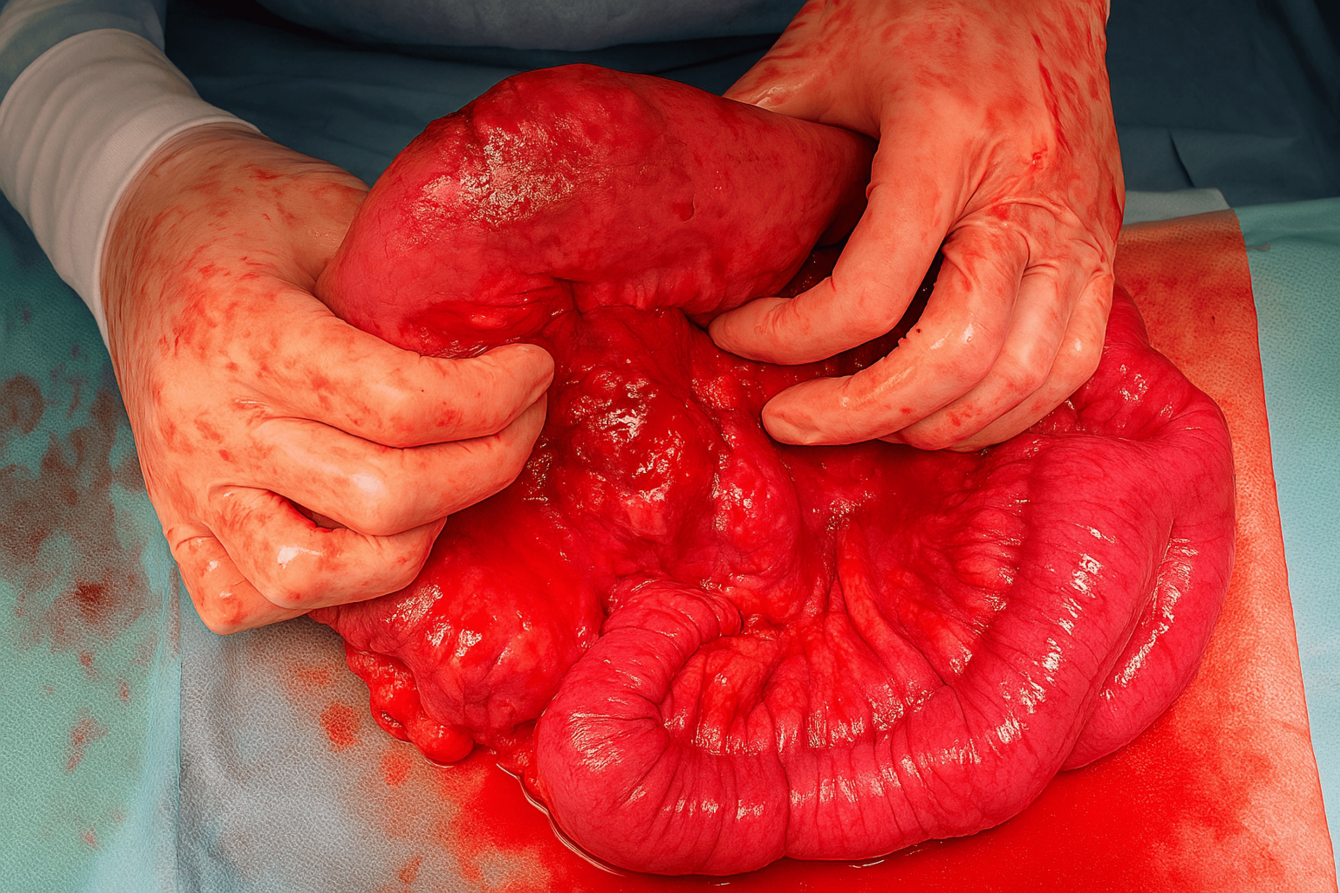
Ileal mass identified intraoperatively.

Based on these findings, an exploratory laparoscopy was proposed for diagnostic assessment and treatment. Intraoperatively, an extensive adhesive peritonitis was encountered, with severe thickening and global inflammation of the small bowel, preventing the identification of any lesions. Surgery was converted to laparotomy, and a mass on the antimesenteric border of the terminal ileum was found, with no bowel perforation (Figure 2). A segmental resection was conducted, involving the lesion and the surrounding thickened bowel, in a total resected area of 20 cm. Primary anastomosis was carried out.

Postoperatively, he received broad-spectrum antibiotics, with ceftriaxone and metronidazole for seven days, and parenteral nutrition. During this period, the patient maintained significant abdominal drainage, summing approximately 500 mL per day of seropurulent fluid. The patient developed profuse sweating requiring bed linen to be changed twice daily. On day 7 postoperatively, a sudden clinical deterioration was noted, with severe aggravation of the intensity of abdominal pain, which was persistent and did not respond to intravenous medication and affected all abdominal area without radiation, together with enteral content drainage suggestive of anastomotic leakage. On examination, the patient appeared pale and diaphoretic, with tachycardia and evidence of poor peripheral perfusion despite a normal blood pressure. Urine output remained normal. An emergent laparotomy was carried out, where a partial anastomotic dehiscence was identified. New bowel resection and anastomosis were conducted. Peritoneal fluid was sent for microbiological and cytological analysis. Broad-spectrum antibiotic prophylaxis with piperacillin-tazobactam was initiated.

Two days after the second surgery, the patient’s condition rapidly deteriorated, with fever, productive cough, and acute respiratory distress requiring supplemental oxygen (2 L/minute). Chest radiography showed no consolidation, and diffuse infiltrates were seen bilaterally; no effusion was found. Laboratory tests demonstrated new-onset normocytic, normochromic anemia; leukocytosis with marked neutrophilia; and significant elevation in lactate dehydrogenase and CRP (Table 1). The patient also had metabolic acidosis (pH, 7.19; pCO_2_, 42; pO_2_, 62; HCO_3_, 16; and base excess {BE}, -9.6), but ions were stable under calcium and potassium supplementation through intravenous fluids. Microbiological studies, including blood, urine, and stool cultures, remained negative.

**Table 1:** Laboratory results at ICU admission. ICU, intensive care unit; LDH, lactate dehydrogenase

Laboratory parameter	ICU admission	Reference range	Interpretation
Hemoglobin (g/L)	88	120-160	↓
White blood cell count (×10⁹/L)	13.31	4.50-11.00	↑
Neutrophils (×10⁹/L)	11.65	1.80-7.70	↑↑
C-reactive protein (mg/L)	470	0.0-5.0	↑↑↑
LDH (U/L)	925	135-225	↑↑
Aspartate aminotransferase (U/L)	50	10-34	↑
Alanine aminotransferase (U/L)	50	10-44	↑
Creatinine (mg/dL)	0.8	0.7-1.2	Normal
Blood urea nitrogen (mg/dL)	10	5-18	Normal
Sodium (mmol/L)	143	135-145	Normal
Potassium (mmol/L)	3.6	3.5-5.0	Normal
Chloride (mmol/L)	98	95-105	Normal

**Figure 3:**
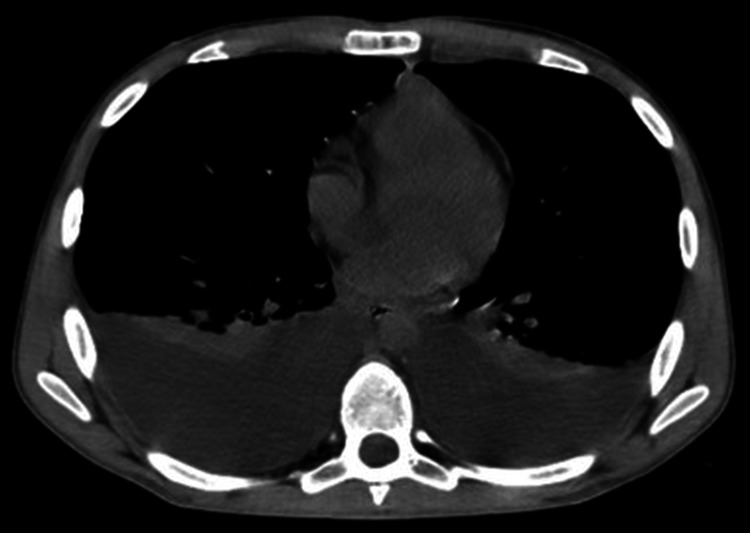
Thoracic CT: bilateral pleural effusion. CT: computed tomography

**Figure 4:**
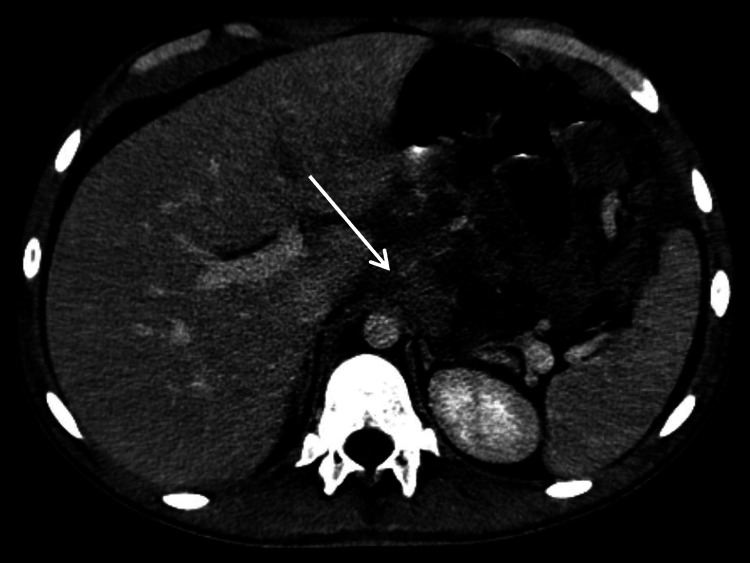
Abdominopelvic CT demonstrating multiple enlarged lumbar-aortic and mesenteric lymph nodes. The arrow indicates confluent adenopathic clusters surrounding the celiac trunk, suggestive of lymphoproliferative involvement. CT: computed tomography

Given the ongoing clinical and laboratory deterioration, thoraco-abdominopelvic computed tomography (CT) scan was performed a few hours later, showing moderate to large bilateral pleural effusion (Figure 3); multiple confluent lymphadenopathies (cardiophrenic angles/pericardial fat, mesenteric and celiac trunk, and lumbar-aortic and para-aortic regions, Figure 4); the thickening of the mesentery, peritoneum, diaphragmatic surfaces, greater omentum, and pleura, suggestive of lymphoproliferative disease; and small to moderate ascites and hepatomegaly (18.3 cm).

Given these findings and the clinical deterioration, the patient was transferred to the pediatric intensive care unit (ICU). Standard infectious disease screening (including viral serologies and Quantiferon) was performed, all of which were negative. During hospitalization, the patient also developed marked unintentional weight loss (11% of body weight).

Diagnostic thoracentesis with immunophenotyping of the pleural effusion revealed immunoglobulin heavy chain (*IGH*) gene (14q32.33) and myelocytomatosis (*MYC*) oncogene (8q24.21) rearrangements, consistent with BL. The immunophenotyping of peritoneal fluid obtained during the second surgery also demonstrated involvement by mature B-cell lymphoproliferative disease. No evidence of disease was identified in peripheral blood, bone marrow aspirate, and biopsy.

Histopathological reports from both surgical specimens were available after the patient had started chemotherapy and confirmed a peripheral B-cell lymphoma with features of BL. Epstein-Barr virus (EBV) in situ hybridization was negative.

The patient started chemotherapy according to the Inter-B-NHL Ritux 2010 pediatric protocol. On day 1, he received intrathecal methotrexate and hydrocortisone, and systemic treatment with methylprednisolone 30 mg/m² every 12 hours was initiated. On day 4, vincristine 1 mg/m² and cyclophosphamide 300 mg/m² were administered. Tumor lysis syndrome prophylaxis followed protocol recommendations and included rasburicase 7.5 mg/day and intensive intravenous hydration (3 L/m²/day) from the first day of chemotherapy. After this initial phase, he was transferred to a pediatric oncology center, where he continued multi-agent intensive chemotherapy according to the Inter-B-NHL Ritux 2010 protocol. During the first cycles at the oncology center, treatment was complicated only by mild toxicity, namely, grade I oral mucositis and perineal candidiasis, which resolved with appropriate topical and supportive care. A follow-up thoraco-abdominopelvic CT performed approximately one month after treatment initiation showed overall radiological improvement, with a marked reduction of peritoneal and omental thickening and lymphadenopathy. He showed rapid clinical improvement, with the resolution of abdominal pain and constitutional symptoms during the initial treatment phase, and remains under follow-up at the pediatric oncology center.

## Discussion

This case highlights a rare and clinically important presentation of Burkitt lymphoma in an adolescent who developed acute abdominal pain with rapid clinical deterioration, mimicking a perforated Meckel’s diverticulum with peritonitis. Although acute abdomen is a frequent complaint in teenagers, neoplastic causes must not be overlooked, as they remain part of the differential diagnosis and early recognition significantly influences survival and reduces complications. Burkitt lymphoma, in particular, is characterized by extremely rapid proliferation and aggressive systemic spread, and in this patient, the diagnosis was only established once widespread disease was already present. This case underscores the need for heightened clinical suspicion in atypical or rapidly evolving presentations, even when they resemble more common surgical emergencies.

BL is one of the most aggressive subtypes, representing about 30%-50% of pediatric non-Hodgkin lymphomas. It is characterized by extranodal involvement, rapid proliferation (tumor doubling time as short as 24-48 hours), and frequent acute complications [5,6]. In Western countries, the sporadic form predominates, typically involving the ileocecal region and mesentery and commonly presenting with abdominal pain, distension, or bowel obstruction [2]. In some cases, the initial manifestation may be subtle, with misleading symptoms, delaying the diagnosis.

We report a case of a previously healthy adolescent who presented with urinary symptoms, followed by an acute abdomen, which delayed the diagnosis of the underlying hematologic malignancy. The urinary complaints were most likely secondary to extrinsic compression from the intra-abdominal tumor [7]. Given that urinary tract infections are uncommon in otherwise healthy adolescent boys with no active sexual life, such a presentation should raise suspicion for alternative etiologies, including malignancy, particularly when there is no clinical improvement after initial treatment.

The patient’s symptoms rapidly progressed to an acute abdomen, with ultrasonography suggesting Meckel’s diverticulum complicated by peritonitis. Meckel’s diverticulum is the most common congenital anomaly of the gastrointestinal tract, affecting 2% of the population [8,9], and is usually asymptomatic, though it may mimic appendicitis or develop a diverticulitis [10,11]. Burkitt lymphomas mimicking Meckel’s diverticulitis are rare, with only a few isolated cases reported in the literature [11,12]. Distinguishing Burkitt lymphoma from Meckel’s diverticulum can be challenging in acute presentations, but several demographic and clinical features may help guide suspicion. Burkitt lymphoma typically affects children and adolescents, most often between five and 15 years old, with a slight male predominance and, depending on the subtype, higher incidence in individuals of African descent, whereas Meckel’s diverticulum is a congenital anomaly affecting all ethnicities, with symptomatic cases more common in young boys. Clinically, Meckel’s diverticulum usually presents with painless rectal bleeding, diverticulitis-like pain, or signs of perforation or obstruction, whereas Burkitt lymphoma more often causes progressive abdominal pain, distension, weight loss, vomiting, or features of bowel obstruction due to rapidly enlarging masses. On examination, Meckel’s diverticulum may produce localized peritoneal signs if inflamed or perforated, while Burkitt lymphoma often reveals a palpable abdominal mass, generalized tenderness, hepatosplenomegaly, or evidence of systemic compromise. Rapid clinical decline, systemic symptoms, or the presence of a firm abdominal mass should therefore raise concern for an underlying lymphoproliferative disorder rather than a primary surgical pathology.

Abdominal Burkitt lymphoma typically arises in the ileocecal region [8]. While abdominal involvement is common, emergency presentations such as acute appendicitis, peritonitis, or intussusception remain rare [3,9,13]. Our case illustrates how BL may masquerade as a primary surgical disease. This diagnostic pitfall is clinically relevant, since surgical procedures may delay the correct diagnosis and initiation of oncologic therapy, while also increasing postoperative morbidity. Awareness that BL can have this sort of presentation may help clinicians maintain a broader differential diagnosis, particularly when abdominal findings are unusually severe or do not fit the expected pattern.

In this case, the diagnosis was achieved not from the resected mass but through the cytological analysis of peritoneal and pleural effusions, which rapidly identified malignant lymphoid cells with *MYC* translocation. The diagnosis was delayed because the histological analysis of the surgical specimen was not immediately available, as it required processing in an external pathology laboratory, preventing an early and definitive identification of Burkitt lymphoma before the patient’s clinical deterioration. Despite this limitation, the rapid turnaround of in-house molecular studies performed on the pleural fluid allowed for the timely confirmation of the diagnosis, even before the histopathology results were issued. This underscores the crucial role of expedited molecular testing in acute presentations, particularly when conventional histology is delayed, as early diagnostic clarification directly influences management and outcomes in such an aggressive malignancy.

Diagnostic delay in BL has significant prognostic implications. It is a rapidly proliferating tumor, and even short delays in initiating therapy may result in rapid clinical deterioration [6,14]. Burkitt lymphoma is highly responsive to short intensive chemotherapy regimens, with survival rates exceeding 80% in pediatric and adolescent populations when promptly treated [15,16].

This case reinforces the need for multidisciplinary management and the awareness of atypical BL presentations. For adolescents presenting with an acute abdomen, especially when associated with systemic inflammatory response and unexplained imaging findings, hematologic malignancy should remain included in the differential diagnosis.

## Conclusions

This case highlights key clinical lessons: Urinary symptoms in adolescent men warrant careful evaluation beyond simple infection, Burkitt lymphoma should be considered as a potential mimicker of common surgical emergencies, and effusion cytology can provide a rapid pathway to diagnosis, enabling the earlier initiation of therapy. Ultimately, a high index of suspicion and a multidisciplinary approach are essential when managing adolescents with atypical or rapidly evolving abdominal presentations.

Our case emphasizes the importance of including lymphoproliferative disorders in the differential diagnosis of acute abdomen in adolescents. Owing to the tumor’s rapid proliferation and high chemosensitivity, the early diagnosis and prompt initiation of chemotherapy are essential to optimize outcomes.
